# Translating into Practice Cancer Patients’ Views on Do-Not-Resuscitate Decision-Making

**DOI:** 10.3390/cancers8100089

**Published:** 2016-09-27

**Authors:** Ian N. Olver, Jaklin A. Eliott

**Affiliations:** 1 Sansom Institute, University of South Australia, Adelaide 5000, Australia; 2 School of Public Health, University of Adelaide, Adelaide 5000, Australia; jaklin.eliott@adelaide.edu.au

**Keywords:** do-not resuscitate, advance care directive, cancer, DNR policy

## Abstract

Do-not-resuscitate (DNR) orders are necessary if resuscitation, the default option in hospitals, should be avoided because a patient is known to be dying and attempted resuscitation would be inappropriate. To avoid inappropriate resuscitation at night, if no DNR order has been recorded, after-hours medical staff are often asked to have a DNR discussion with patients whose condition is deteriorating, but with whom they are unfamiliar. Participants in two qualitative studies of cancer patients’ views on how to present DNR discussions recognized that such patients are at different stages of understanding of their situation and may not be ready for a DNR discussion; therefore, a one-policy-fits-all approach was thought to be inappropriate. To formulate a policy that incorporates the patient’s views, we propose that a standard form which mandates a DNR discussion is replaced by a “blank sheet” with instructions to record the progress of the discussion with the patient, and a medical recommendation for a DNR decision to guide the nursing staff in case of a cardiac arrest. Such an advance care directive would have to honor specifically expressed patient or guardian wishes whilst allowing for flexibility, yet would direct nurses or other staff so that they can avoid inappropriate cardiopulmonary resuscitation of a patient dying of cancer.

## 1. Introduction

It is the evening nursing shift in a large urban hospital. A nurse notices that one of her elderly patients with metastatic cancer who is receiving palliative treatment has deteriorated over the 16 h since her last shift. She consults the case records and discovers that there has been no do-not-resuscitate (DNR) order written on his chart. She asks the resident medical officer on call to speak with the patient so a DNR order can be recorded before the night-duty staff take over.

The default policy in many hospitals in the developed world is for nurses to call for cardiopulmonary resuscitation on a patient in the absence of a specific instruction to not attempt cardiopulmonary resuscitation [1]. Nursing staff do not have the authority to override this even if, in their judgment, a resuscitation attempt would be inappropriate or futile. This is distressing since a patient whose heart stops as part of natural dying of a disease such as cancer should be differentiated from one who suffers a sudden unexpected cardiac event [2]. Given that less than 20% of patients are discharged from the hospital after attempted cardiopulmonary resuscitation, the harm of the intervention can often outweigh the potential benefit. As a result, “Do Not Attempt Cardiopulmonary Resuscitation (DNACPR)”, often called “Do Not Resuscitate (DNR)”, decisions are documented and implemented [3].

International guidelines recommend that end-of-life discussions should occur early after the diagnosis of an advanced cancer [4]. Documentation of an Advance Care Plan (ACP) enables patients to make decisions, often in consultation with their clinicians and families, about their future healthcare [5]. These can be useful if patients subsequently lose the capacity to make those decisions [6].

The issue is whether discussing a DNR order with a patient early in the course of an advanced cancer or at a time dictated by admission to hospital may be a distressing experience for patients who either (a) have not yet accepted that they are dying, or (b) have accepted that there is no further active treatment for their condition, but find specific discussions on cardiopulmonary resuscitation during a specific admission to hospital distressing, as they may interpret this as the medical staff pronouncing that death is imminent.

Having performed two qualitative studies to ascertain the opinion of patients with cancer about their preferences for how and when DNR discussions occur, we then sought to formulate policy based on that input and illustrate how it would translate into practice by using clinical scenarios [7,8].

## 2. Prior Studies

In two separate qualitative studies approved by the Royal Adelaide Hospital Ethics Committee we ascertained patients’ views on the processes of end-of-life decision-making through discourse analysis of interviews [7,8]. Both patient groups, with the sample size determined by data saturation, had received diagnoses of cancer, but the first group of 23 were not considered near the end of their lives, whereas the second group of 28 patients were considered to be within weeks of death by their treating practitioners.

A clinical psychologist conducted interviews with the patients in the first study, and a social scientist conducted those in the second study. Demographic details were recorded and open-ended questions elicited comments on who should make DNR orders and what information they would need, when they should be made in the course of an illness, whether they should be recorded and who should be involved.

Interviews were transcribed and data were sorted into categories, with concept-based categories guided by thematic analytical techniques [9]. Speech items representing particular themes were noted and examined for relevance across interviews. The discourse analytic technique was used to examine these items for diversity or consistency between or within speakers [10].

These discourse analyses created a rich dataset from which we have published several insights, but the two summary papers exploring patients’ perceptions of do-not-resuscitate decision-making enabled us to formulate a policy based on the patients’ views [7,8].

Most patients in both studies assessed DNR discussions as appropriate, based on the current medical condition and a poor prognosis, and agreed that, in the event of the heart stopping, the patient should not be “*brought back to life*”. Many perceived resuscitation as interfering with a “*natural*” process where the patient would “*go quietly*”. They saw a DNR decision as their decision as autonomous adults, but needed medical facts about their condition, and the likelihood of the success of resuscitation, to make a rational decision. They favored having the time to discuss their decision with medical staff and family, and recognized that this decision could change over time and altered circumstances, summarized by one patient as “…*getting all sides and having a chance to talk it over with other people...the more time they’ve had to think about it, and weigh up and work out what’s going to be best for them, for families and so on, is much better…*”.

Patients were ambivalent about when DNR orders should be discussed so as to avoid distressing a patient who may “*not handle it well*” or “*go into shock*” and they thought that this was most appropriately judged by a doctor with experience in such situations; one patient spoke of waiting for the doctor’s cue: “*Oh well, if he is going to see me in a month’s time, that’s a month I have got to make sure I’m here, do you see (laughing), so if he came in one day and says “well there’s nothing more we can do, don’t bother coming back” that’s the time to worry.*” In general, they favored a written record of the decision to ensure that their wishes were communicated but also accepted the need for substituted decision-making if patients were no longer competent, and most often designated the family as appropriate for that role. The patients interviewed recognized that a one-policy-fits-all approach to the timing and extent of the discussion could be harmful as each patient would be at a different stage of their acceptance of their situation.

## 3. Recommendations for DNR Policy

In the ideal situation, and respecting the importance of individual patient autonomy, a DNR decision and an ACP would be mutually agreed upon between the doctor and a competent patient based on the prognosis and the goals of treatment. This process protects patients at the end of life against intrusive treatments that they may not want or which are medically judged as futile.

The policy for DNR decision-making also needs to respect the wishes of the patient who does not wish to have such a discussion at a particular time or whom their clinician assesses as not ready for such a discussion. However, even in that situation there is a need to solve the problem of what instructions to leave nursing staff so they do not have to call for cardiopulmonary resuscitation inappropriately if someone in this group is naturally progressing towards death.

Based upon the patients’ views in our studies, a form for recording the instructions on DNR should be flexible enough to accommodate that spectrum of possibilities. We propose that the advance care directive be a “blank sheet” prominently placed in the case notes, prefaced by instructions for the medical staff about how to complete it in a variety of scenarios. The sheet would be accompanied by instructions as to what to record but there would be no common format for what was recorded, thus allowing a narrative reflecting each individual patient’s circumstances and a medical directive to nursing staff based on that individual’s circumstances. Accordingly, on the sheet, a patient’s senior treating doctor would record a narrative of where their ongoing discussions with their patient about prognosis and treatment options have reached. If these discussions have not specifically arrived at a DNR decision, then a recorded medical directive on the use, appropriateness, or futility of resuscitation would be recorded to provide guidance to nursing staff.

This sheet would be dated and updated as circumstances changed. There would be a provision for the patient, patient’s doctor, nurse, or (after discussion with the patient) a relative to sign if that was thought helpful as part of good communication.

To illustrate what instruction could be provided for completing the DNR sheet, examples from “*Guidelines for an Advance Care Plan*” found in the Clinical Practice Manual of the Royal Adelaide Hospital are provided. They were based on the South Australian Statement on Withdrawing and Withholding Treatment, Consent to Medical Treatment and Palliative Care Act 1995, and the Guardianship and Administration Act 1993, now superseded by the Advance Care Directive Act 2013 [11].

The “blank sheet” would be accompanied by instructions on the rationale for documentation of DNR, and examples of what could be recorded on it in various circumstances. Case examples of what would be recorded follow.

### 3.1. Case 1

An ACD including DNR orders has been discussed with the patient and family.

In view of Mr. Smith’s advanced lung cancer with liver and bone metastases, in the event of cardiac or respiratory arrest, cardio-pulmonary resuscitation would be inappropriate and should not be commenced. Mr. Smith and his family are aware of the terminal nature of his illness and agree that treatment should be directed at comfort measures only. Cardio-pulmonary resuscitation has been specifically discussed with them. Mr. Smith’s treatment plans are directed at pain relief and maintaining hydration.

### 3.2. Case 2

As part of ongoing communication between the patient and her doctor, the patient understands that there is no further active treatment and that she is dying, but a DNR order has not been specifically discussed because the doctor’s judgment is that it would cause great distress at this specific time.

The prognosis has been discussed with the patient who understands that no further active treatment is available and that the goal of treatment is palliation. No specific discussion about a Do Not Resuscitate order was considered appropriate at the stage the discussion reached. The medical recommendation is that the patient’s treatment includes full medical measures including transfusion and antibiotics but in the event of cardiac arrest no cardio-pulmonary resuscitation is instituted.

### 3.3. Case 3

The patient refuses to enter into a discussion on Do Not Resuscitate and has no relatives.

The patient, who has widespread metastases from cancer, has indicated that she does not want to discuss or document issues of resuscitation but has indicated that she will accept appropriate medical decisions. Since she is dying and no treatment will reverse this situation, care will be limited to symptomatic treatment only and in the event of a cardiac arrest, cardio-pulmonary resuscitation should not be attempted. Mrs. Thomas has no known relatives.

### 3.4. Case 4

A patient in the intensive care unit (ICU) has irreversible global anoxic encephalopathy after a cardiac arrest and the plan is to withdraw medically futile support.

ICU support (e.g., mechanical ventilation, inotropic support) is likely to be withdrawn if continued support is considered to be futile in terms of a good neurologic recovery by 48–72 h post-arrest. In view of the patient’s poor neurologic prognosis following his cardiac arrest, cardio-pulmonary resuscitation should not be undertaken in the event of further cardiac arrest whilst in ICU. The patient’s wife and family are aware of the likely outcome and agree with this recommendation.

## 4. Discussion

An international survey of DNR decision-making which included 43 countries found that 88% had a DNR process but most reported that in practice, resuscitation wishes were discussed with the patients only at least half of the time. Cultural attitudes toward death and the role in society of family were found to influence DNR decision-making [12]. In some countries (e.g., the UK, after the Tracey Judgment) it is now illegal to make DNR decisions without informing the patients [13].

In a systematic review of the barriers and facilitators of DNR decision-making, doctors reported that barriers included a perceived resistance from the patient, a desire not to cause distress and poor health (where patients may be expected to die) [14]. In our study the patients recognized that these barriers were real and needed to be accommodated, but, as has been reported, patients want to be involved in DNR discussions [15].

Facilitators which promote DNR discussion include making it part of routine overall treatment discussions, and training doctors about communication at the end of life [14]. Introducing a standardized recording sheet has also been found to improve the quality of DNR documentation [16]. In our proposal we recommend that the senior clinician who has developed a relationship with the patient must be the doctor who has the DNR discussion. The nature and outcomes of these discussions then can be placed into the context of an overall treatment discussion by the clinicians who have a long-term relationship with the patients.

The doctor is in the best position to judge the likely outcome of cardiopulmonary resuscitation (CPR), notwithstanding that the patients’ decisions often involve more than medical outcomes, and include value judgments [17]. In addition, within Australia, from the legal perspective, it has been accepted that patients cannot demand futile treatments [18]. Furthermore, a doctor (invoking therapeutic privilege) may judge that at a particular time it would be distressing for a patient to engage in a DNR discussion; at worst, this would be regarded as weak paternalism because it is delaying rather than overriding the patient’s decision-making [19].

The utility of the “blank sheet” is that it is a formalized process with instructions, but accommodates both patients who want to express their wishes, and those who do not; documents the medical view of futility or otherwise of DNR; and leaves adequate instruction for the nursing staff even when a patient does not wish to engage in the discussion. That is, it satisfies the needs of the hospital staff without forcing patients into a one-policy-fits-all situation. The default option of resuscitation would still remain if no blank sheet was completed.

In Australian state jurisdictions, legally refusing or withdrawing certain treatments in situations involving a palliative setting or advanced chronic disease is considered acceptable [20]. A competent patient can make the decision and nominate a medical agent, or make an Anticipatory Direction in advance of not being capable of making the decision. A Guardianship Board can also be appointed as a legal guardian if a patient is no longer competent. In these cases, the agent, the Anticipatory Decision, or the Guardianship Board must be consulted before a medical decision is made, including that which is recorded in any Advance Care Plan.

Most patients in our study reported that their relatives were the most appropriate surrogate decision-makers and suggested that their relatives would know what decision they would want made. It was also clear that participants held that DNR decisions required that patients or surrogates be informed about the chances of cardiopulmonary resuscitation being successful.

However, autonomous decision-making depends on the nature of the decision, and participants in our study were ambivalent about the timing of a DNR discussion because they recognized that there would be differences between patients’ readiness to have a DNR discussion at any one time in their illness [21]. The importance to the patients of timing of a DNR discussion was also found in a study which asked patients to evaluate a question prompt sheet used to encourage doctor/patient discussions about advance care planning [22]. Several participants suggested that the questions were appropriate but not for them now, maybe later.

There are varying reports of harms associated with DNR discussions. A randomized trial of an intervention consisting of a pamphlet and discussion about DNR versus usual management showed that Advance Care Plans were completed earlier with the intervention without adverse effects, but the study may have been underpowered to show differences [23]. Moreover, a large Dutch study showed that patient information leaflets had no effect on how frequently DNR forms were completed [24]. Alternatively, a patient left with a DNR form by a doctor, without discussion and without family present, was reported in the press as being “terrified” [25].

The two qualitative studies of patient preferences for communicating about DNR decisions interviewed predominantly white Australians. Different policies may be required for groups with other ethnic origins or Aboriginal and Torres Strait islanders [26,27].

## 5. Conclusions

Patients interviewed about DNR decision-making to inform DNR policy recognized that the ideal timing of a DNR discussion would differ between patients. Therefore, standardizing the timing and format of such a discussion to fit with hospital culture where resuscitation is the default option may distress patients who are not ready for such a discussion, and/or subject them to inappropriate interventions if they are dying. One solution is to make documentation of the DNR decision flexible, allowing a narrative written by a patient’s doctor and summarizing a patient’s current understanding of treatment choices and prognosis. If a patient is not ready to make a DNR decision, the doctor should leave a medical directive based on a clinical judgment about the futility of resuscitation, clearly providing instruction for nursing staff.
